# Supplementary material for: Duplex-Repair enables highly accurate sequencing, despite DNA damage
Source: Nucleic Acids Res. 2021 Sep 30;50(1):e1. doi: 10.1093/nar/gkab855 (PMC8755016; doi:10.1093/nar/gkab855)
Supplement: gkab855_Supplemental_File [file gkab855_supplemental_file.pdf]

Duplex-Repair enables highly accurate sequencing, despite DNA damage

Kan Xiong[1+], Douglas Shea[1+], Justin Rhoades[1+], Tim Blewett[1], Ruolin Liu[1], Jin Bae[1], Erica Nguyen[1], G. Mike Makrigiorgos[1,2], Todd R. Golub[1,3], Viktor A. Adalsteinsson[1] *

[1]Broad Institute of MIT and Harvard, Cambridge, MA, 02142, USA

[2]Department of Radiation Oncology, Dana-Farber Cancer Institute and Brigham and Women's Hospital, Harvard Medical School, Boston, MA

[3]Department of Pediatric Oncology, Dana-Farber Cancer Institute, Boston, MA, USA

[+]*These authors contributed equally*.
*To whom correspondence should be addressed. Tel: 617-714-7971; E-mail:viktor@broadinstitute.org

Recalibrate capillary electrophoresis traces:

Lengths of synthetic oligonucleotides are confirmed by IDT's mass spectrometry analysis (data not shown). However, the control peak locations reported from raw fragment analysis by using the Peak scanner 2 software differ from the expected positions (**Table S1**); the peak locations of 6-FAM tagged molecules consistently appear as underestimates whereas those with ATTO 550 present as overestimates.

To interpret the capillary electrophoresis data, we decide to recalibrate the peak locations by using a ladder of synthetic oligonucleotides with known lengths. **Equation S1-2** relates the oligonucleotide length to raw peak locations through linear regression.

$y = 1.0381x - 7.681$ Eq. S1

Equation S1. Linear regression of raw fragment analysis peak locations of the 6-FAM-tagged strands. Experimentally determined values for the oligos tagged with 6-FAM in the 100 bp, 90 bp, 80 bp and 70 bp ssDNA controls (**Table S1** oligos e, d, c, b respectively) were used to generate a model that relates actual oligonucleotide length (x) to the fragment analysis readout (y) for 6-FAM substrates (**Fig. S1A**).

$y = 0.9666x + 5.039$ Eq. S2

Equation S2. Linear regression of raw fragment analysis peak locations of the ATTO 550-tagged strands. Experimentally determined values for the oligos tagged with ATTO-550 in the 100 bp, 90 bp, 80 bp and 70 bp ssDNA controls (**Table S1** oligos i, h, g, f respectively) were used to generate a model that relates actual oligonucleotide length (x) to the fragment analysis readout (y) for ATTO-550 substrates (**Fig. S1B**).

Quantification of library conversion efficiency by ddPCR:

To quantify library conversion efficiency, a ddPCR assay was designed to target the flanking adapter regions. Only fragments with successful double ligation were exponentially amplified within the QX200 ddPCR EvaGreen Supermix (Bio-Rad) and thus detected.

ddPCR assay design
Primer 1: CACTCTTTCCCTACACGACG<br>Primer 2: AGTTCAGACGTGTGCTCTTC

A **B**

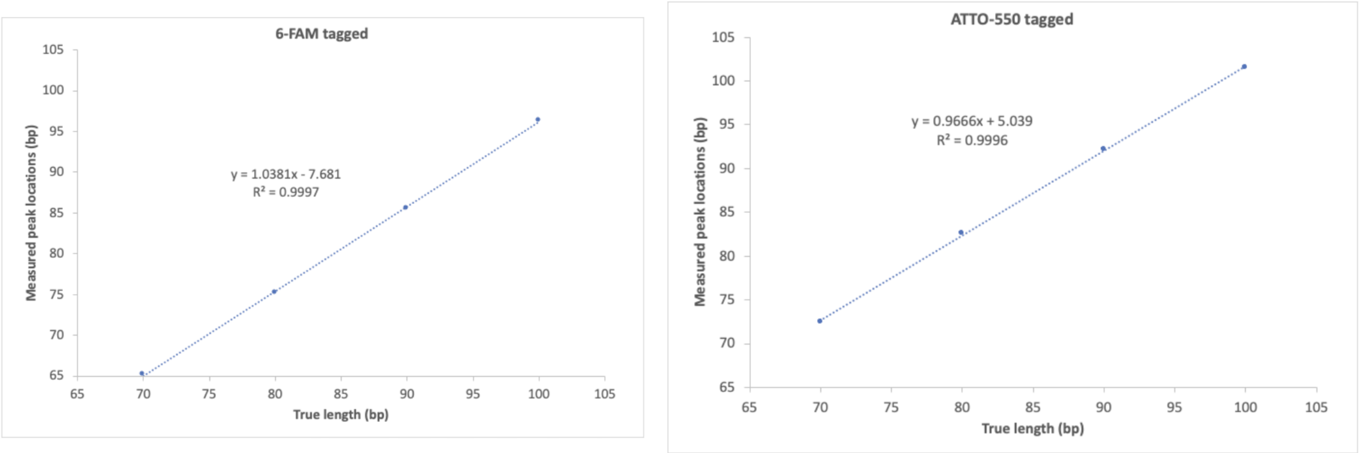

Figure S1: Linear regression of measured capillary electrophoresis peak locations vs. true lengths for (a) 6-FAM-tagged and (b) ATTO-550 tagged oligonucleotides. True lengths of oligonucleotides are confirmed by IDT's mass spectrometry analysis (data not shown).

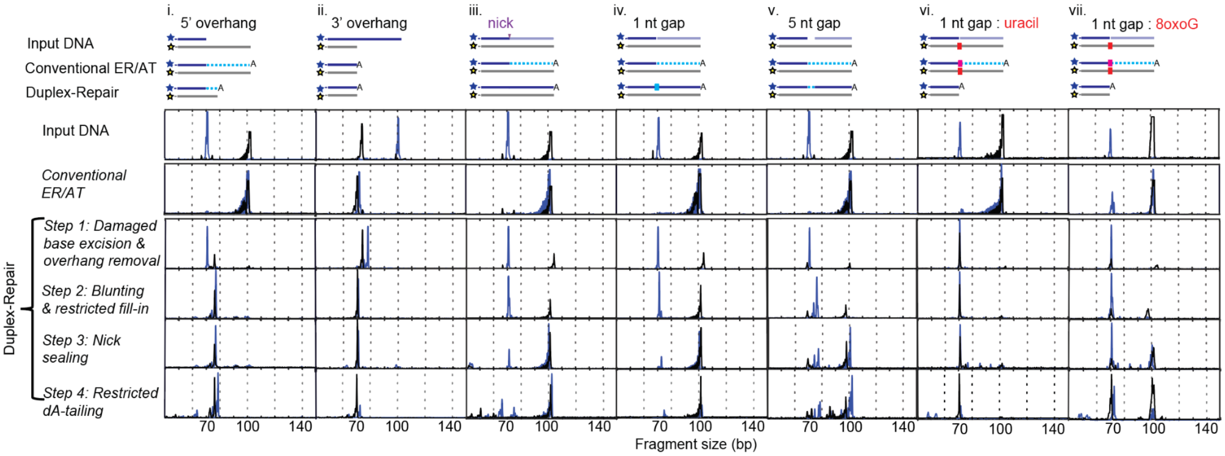

Figure S2: Capillary electrophoresis analysis of synthetic duplexes subjected to each step of Duplex-Repair, versus conventional ER/AT. Each step of duplex repair imparts its intended functionality in producing the intended major product as depicted in Fig. 1 to minimize strand resynthesis seen with Conventional ER/AT. Oligonucleotides with a (i) 5' overhang, (ii) 3'overhang, (iii) nick, (iv) 1 nucleotide gap, (v) 5 nucleotide gap, (vi) uracil across from a 1 nucleotide gap, and (vii) 8oxoG across from a 1 nucleotide gap were subjected to conventional ER/AT and each step of Duplex Repair and sent for capillary electrophoresis. The top strand of each oligonucleotide is labelled with 6-FAM on the 5' end, and the fragment size distributions following each treatment are represented by blue curves. The bottom strand of each oligonucleotide is labelled with ATTO-550 on the 3' end, and the fragment size distributions following each treatment are represented by black curves.

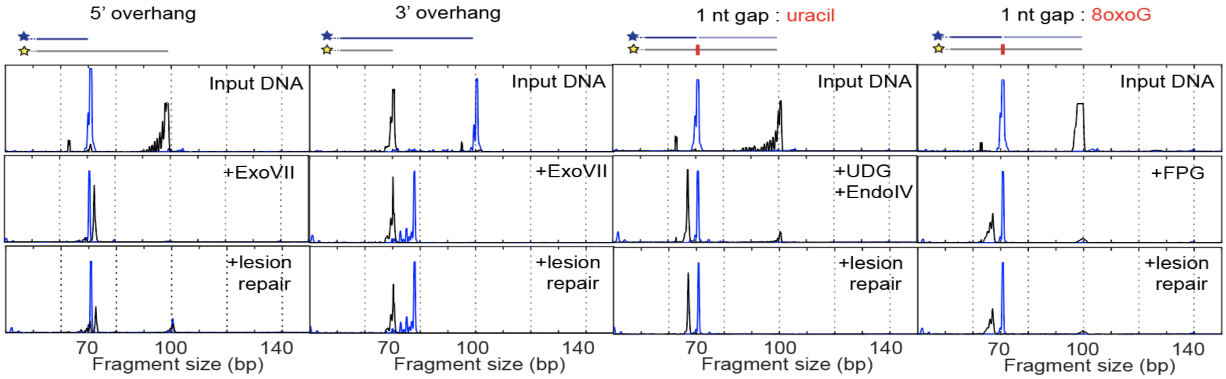

Figure S3: Characterization of the activity of key enzymes in the lesion repair enzyme cocktail by capillary electrophoresis. The activity of key enzymes to rectify each damage motif (middle) is not impacted by other enzymes in the lesion repair enzyme cocktail (bottom). The "lesion repair" condition indicates treatment with Endonuclease IV (EndoIV), Formamidopyrimidine [fapy]-DNA glycosylase (Fpg), Uracil-DNA glycosylase (UDG), T4 pyrimidine DNA glycosylase (T4 PDG), and Endonuclease VIII (EndoVIII), and Exonuclease VII (ExoVII).

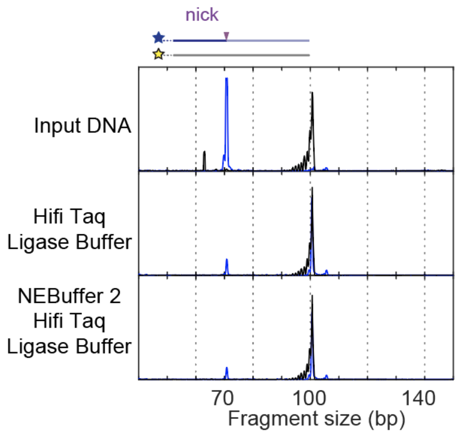

Figure S4: Characterization of the activity of HiFi Taq DNA ligase by capillary electrophoresis. HiFi Taq DNA ligase efficiently seals nicks in NEBuffer 2 and HiFi Taq ligase buffer mix (bottom) as it does in HiFi Taq ligase buffer alone (middle).

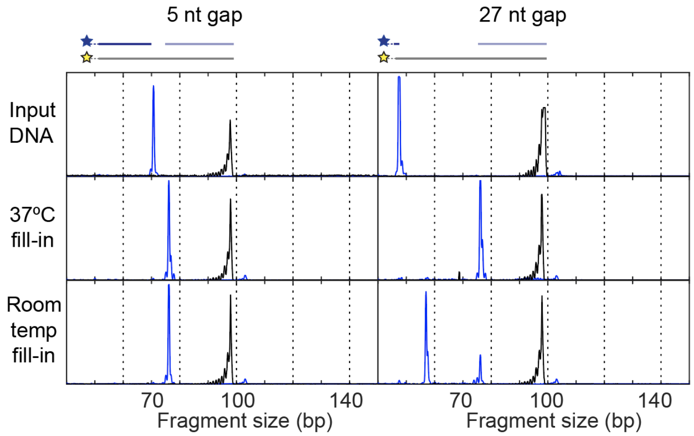

Figure S5: Characterization of the activity of T4 DNA polymerase and T4 polynucleotide kinase by capillary electrophoresis. T4 DNA polymerase efficiently fills in 5 or 27 nt gaps at 37 °C in NEBuffer 2 with no detectable strand-displacement activity (middle). The efficiency of T4 DNA polymerase filling in 27 nt gaps at room temperature, however, is significantly lower (bottom).

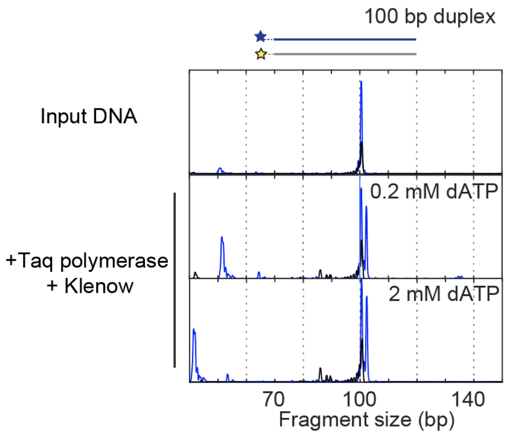

Figure S6: Characterization of the activity of Klenow fragment (exo-) and Taq DNA polymerase by capillary electrophoresis. Klenow (exo-) and Taq DNA polymerase efficiently perform dA-tailing with only dATP present at concentrations of 0.2 mM (middle) or 2 mM (bottom).

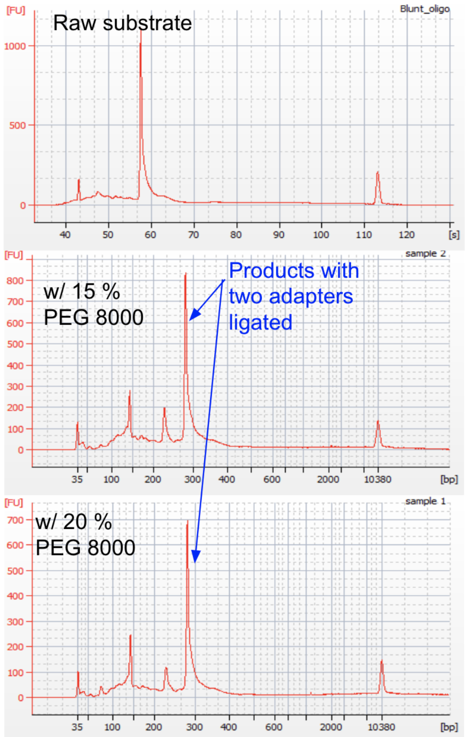

Figure S7: Characterization of the activity of T4 DNA ligase and 5' deadenylase by BioAnalyzer. T4 DNA ligase and 5' deadenylase efficiently ligate NGS adapters to a 166 bp blunted duplex with dA tails in the presence of 15 (top) or 20% (bottom) weight by volume (w/v) PEG 8000. To minimize spurious intermolecular ligation at high PEG concentrations, Duplex-Repair only uses 10% w/v PEG 8000 during adapter ligation. Of note: the unit of the x axis of the top panel could not be converted to bp by BioAnalyzer software.

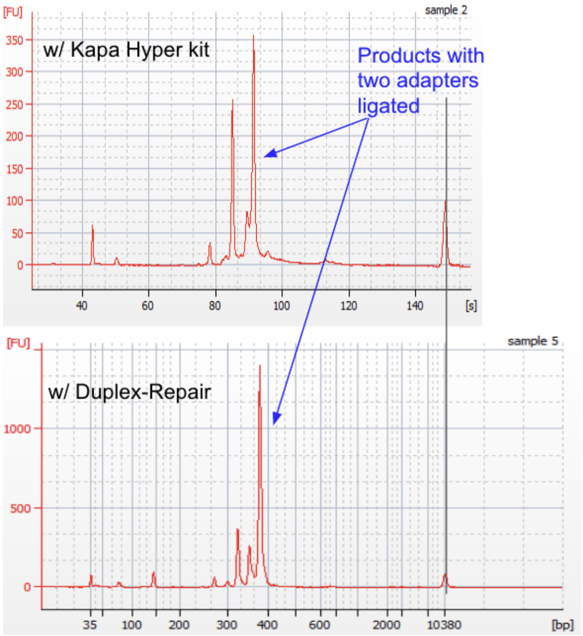

Figure S8: Characterization of the combined efficiency of dA-tailing and adapter ligation by BioAnalyzer. The combined efficiency of dA-tailing and adapter ligation of Duplex-Repair could be higher than that of the Kapa Hyper kit. The input was a 274 bp blunted duplex. Of note, the unit of the x axis of the top panel could not be converted to bp by BioAnalyzer software.

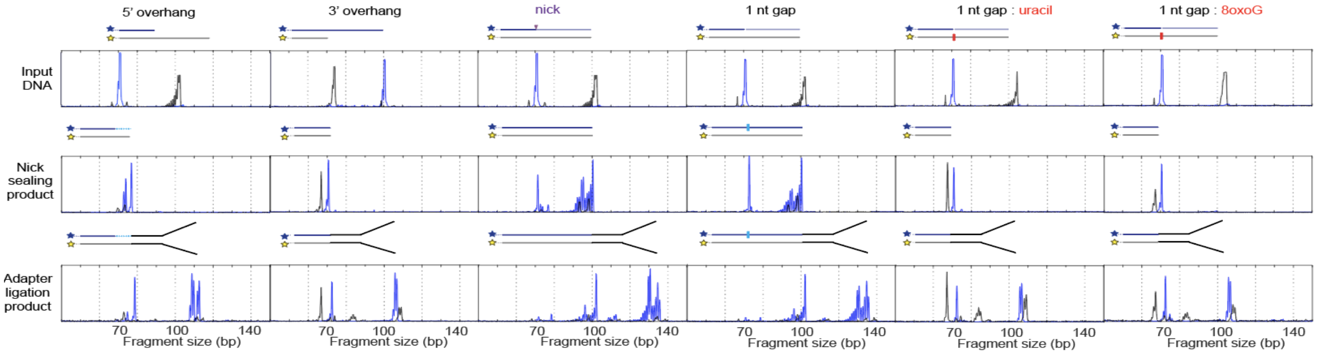

Figure S9: Characterization of the performance of Duplex-Repair (after optimizing reaction conditions and eliminating multiple Ampure cleanups) by capillary electrophoresis. Duplex-Repair facilitates the formation of a major product of NGS adapter-ligated oligonucleotides that are ready for sequencing applications. The 'nick sealing products' (middle) were collected following steps 1-3 of duplex repair but prior to dA-tailing. The 'adapter ligated products' (bottom) have undergone the entire Duplex-Repair protocol and ligation to NGS adapters, which add an additional 39-40 or 37-38 bp (unique molecular indices can be either 3 or 4 base pairs) to the exposed 3' and 5' ends of oligonucleotides after Duplex-Repair respectively (note: adapters in schematic not drone to scale).

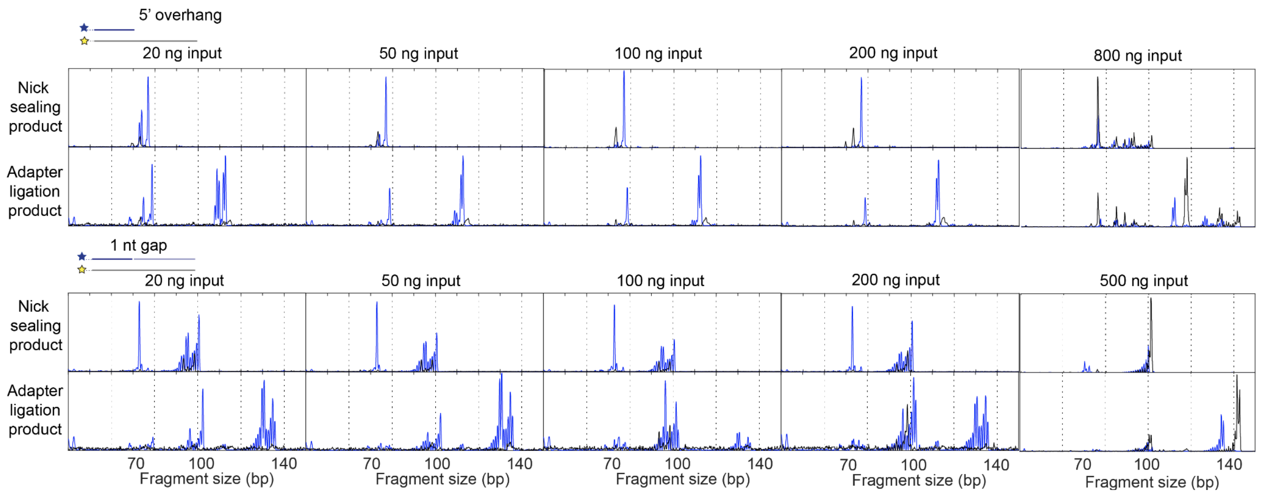

Figure S10: Characterization of the performance of Duplex-Repair (after optimizing reaction conditions and eliminating multiple Ampure cleanups) as a function of DNA input mass by capillary electrophoresis. Duplex-Repair is effective at preparing cfDNA inputs ranging from 20 to 800 ng for NGS. The 'nick sealing products' (top rows) were collected following steps 1-3 of duplex repair but prior to dA-tailing. The 'adapter ligated products' (bottom rows) have undergone the entire Duplex-Repair protocol and ligation to NGS adapters, which add an additional 39-40 or 37-38 bp (unique molecular indices can be either 3 or 4 base pairs) to the exposed 3' and 5' ends of oligonucleotides after Duplex-Repair respectively.

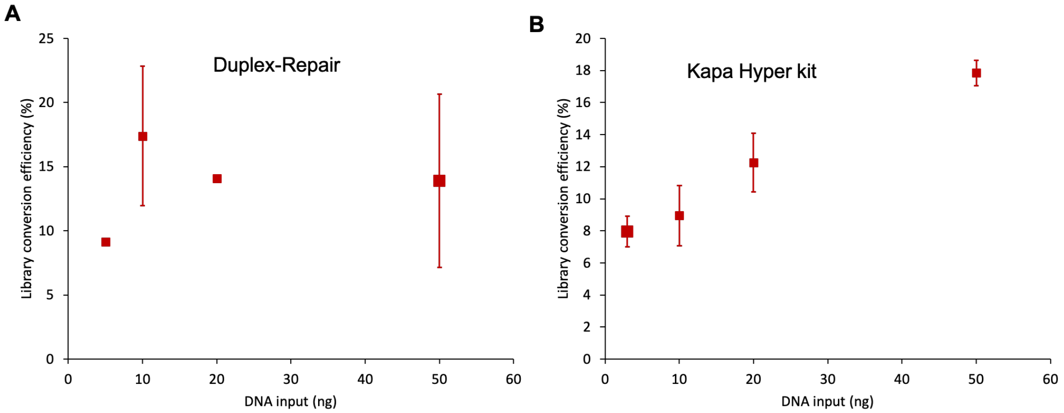

Figure S11: The measured library conversion efficiencies of Duplex-Repair vs. the Kapa Hyper kit as a function of a gDNA input by using a ddPCR assay. The library conversion efficiencies of Duplex-Repair are comparable to this with conventional ER/AT using the Kapa Hyper kit. ddPCR primers used are detailed in the Supplementary Text.

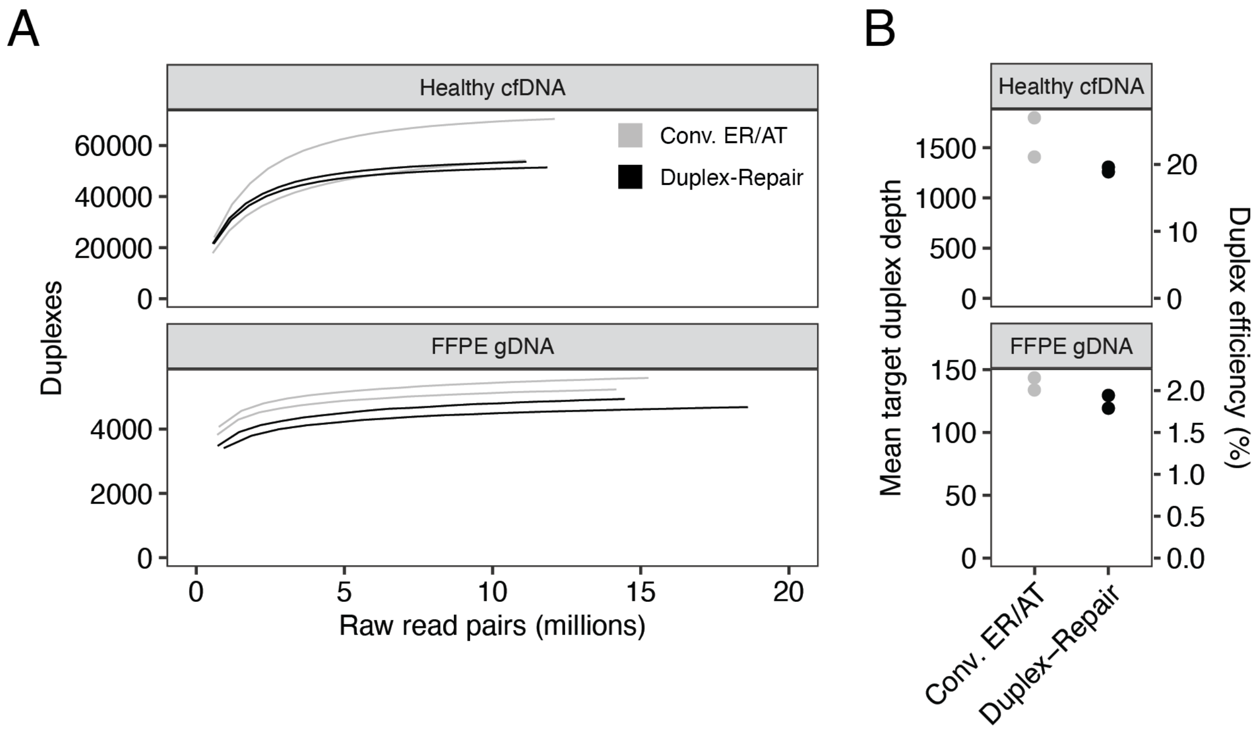

Figure S12: Duplex recovery. (A) Total duplexes recovered for 20 ng input libraries sequenced to saturation with a small hybrid capture panel. (B) Mean duplex depth across target bases and estimated duplex efficiency. Maximum theoretical duplex depth calculated for duplex efficiency estimates assumes one haploid genome equivalent is equal to 3 pg of DNA input.

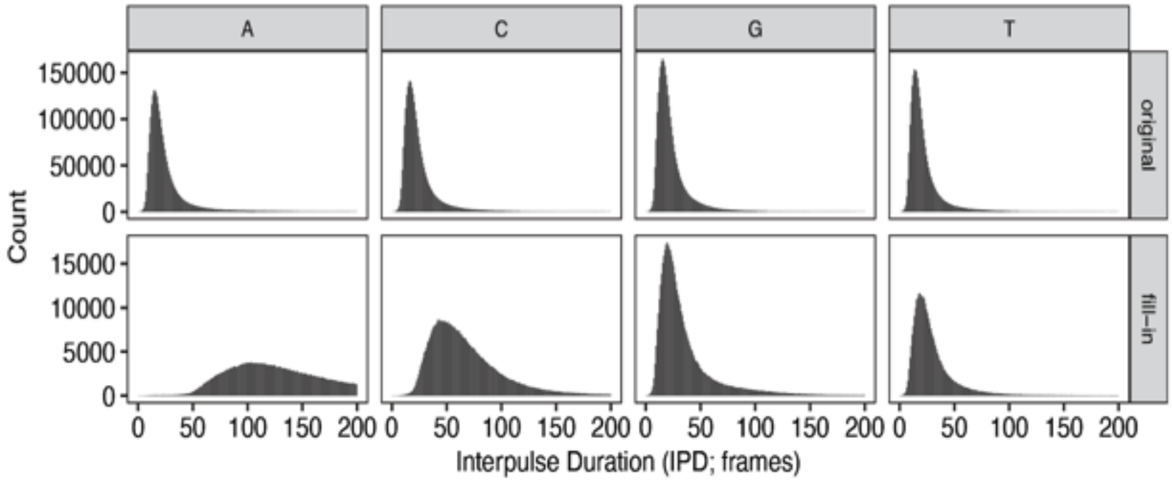

Figure S13: Establishment of an assay for quantifying the number of bases resynthesized during ER/AT. Histogram of aggregate bases and their IPDs, labeled as original or fill-in based on which region of the synthetic oligos they were derived from. Regions that divide original and fill-in regions were avoided for collection.

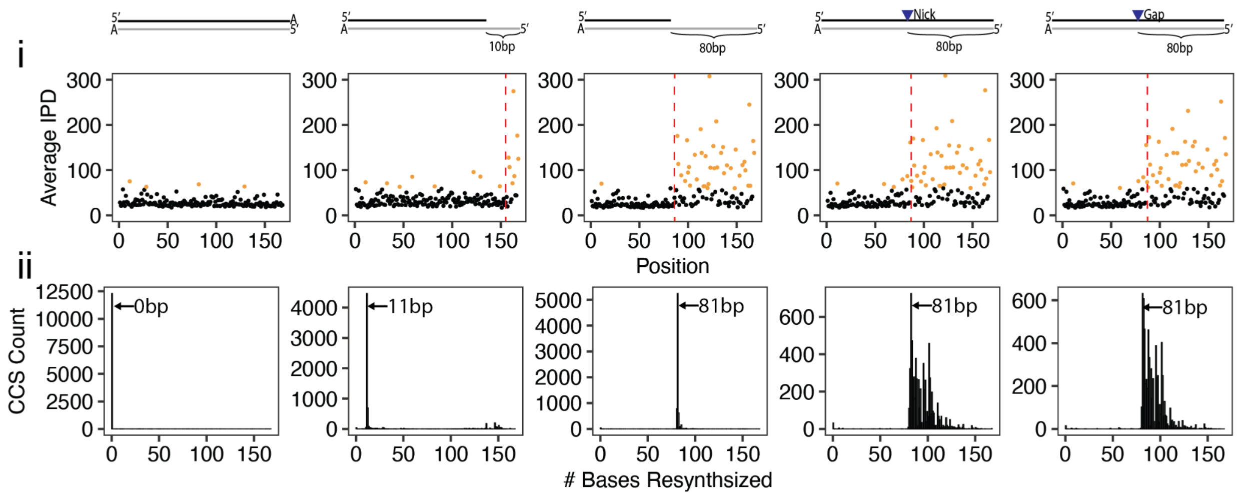

Figure S14: Measured interpulse duration (IPD; in frames) (i) and predicted percentage of bases resynthesized (ii) as a function of the base position on five synthetic oligonucleotides treated with conventional ER/AT and with modified dNTPs. Longer IPDs, colored orange if greater than 60 frames, result from modified bases. Red dashed lines indicate where resynthesis is expected to start during ER/AT.

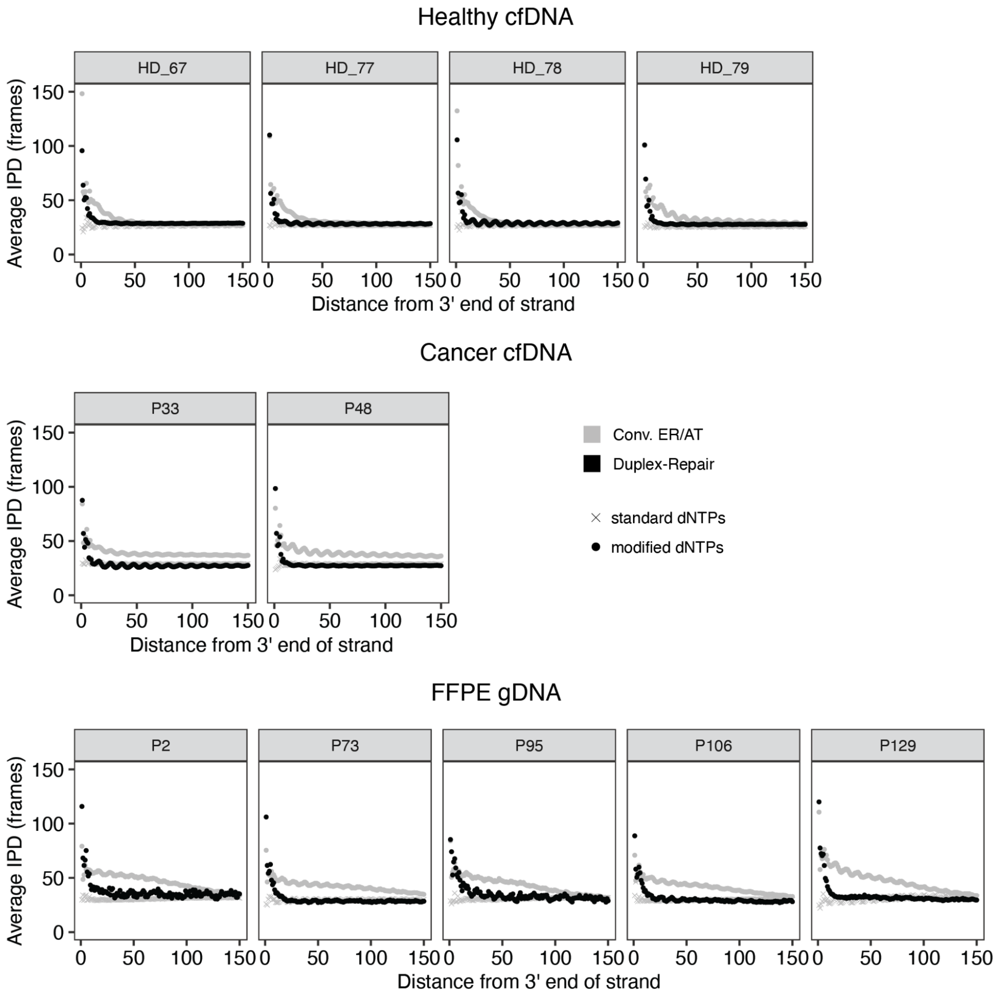

Figure S15: Identification of resynthesized regions by reading out interpulse durations during PacBio SMRT sequencing. Average interpulse durations (IPD; in frames) for each position relative to distance from the end of the original duplex DNA fragment for healthy cfDNA, cancer patient cfDNA, and FFPE tumor biopsies across several individuals.

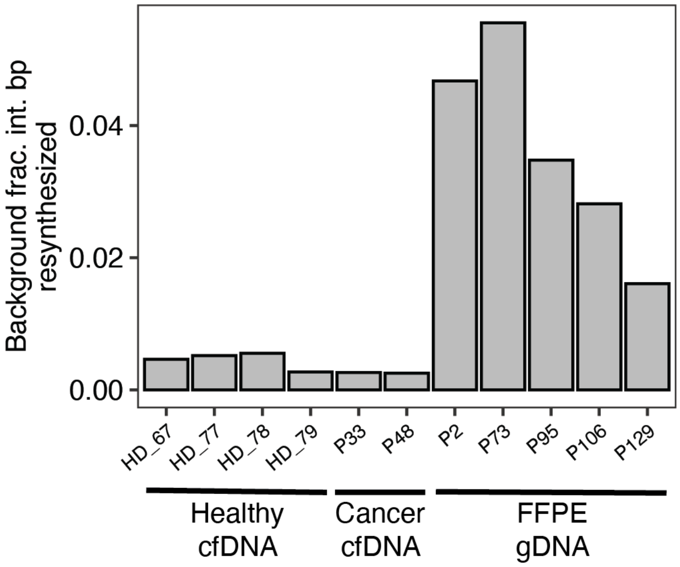

Figure S16: Background estimated resynthesis of interior base pairs using standard dNTPs across FFPE and cfDNA sample types.

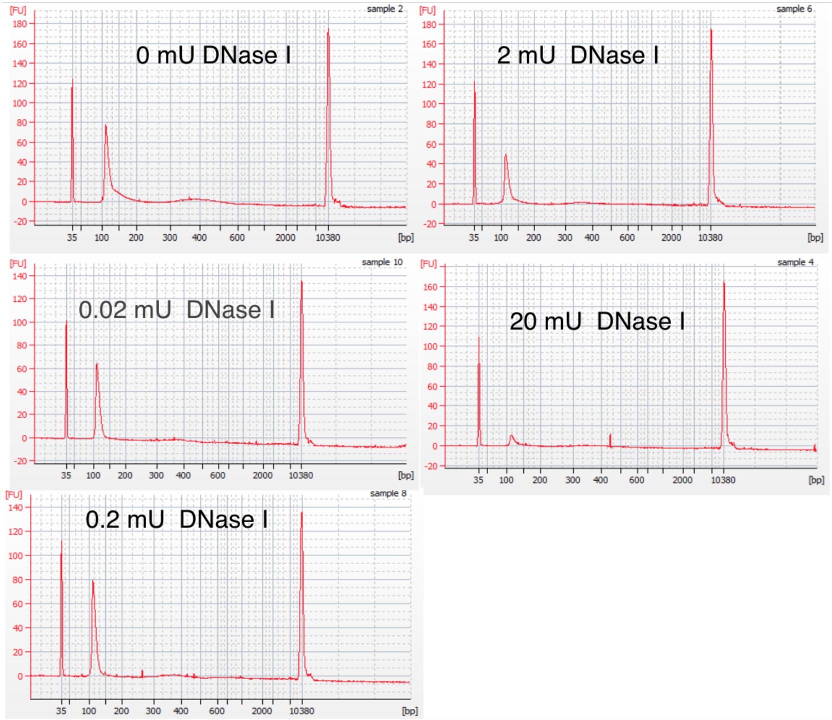

Figure S17: Characterization of the activity of DNase 1 by BioAnalyzer. The input was a 100 bp dsDNA oligo. The results show that up until 20 mU of DNase 1, the dominant fragment length is still 100 bp.

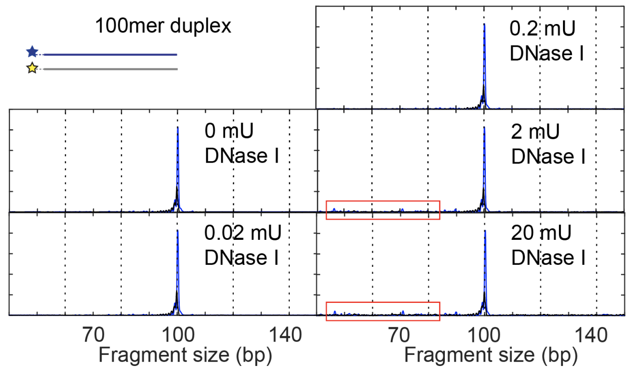

Figure S18: Characterization of the activity of DNase 1 by capillary electrophoresis. For all concentrations of DNAse 1 tested, the major product as determined by capillary electrophoresis is the 100mer duplex. However, intermediate-sized fragments (highlighted in red boxes) are detected with 2 and 20 mU of DNase 1 , suggesting that ≥2 mU of DNase 1 nick but do not significantly degrade dsDNA. These intermediate-sized fragments are present in capillary electrophoresis traces, as heat pretreatment and denaturation is required, but not on BioAnalyzer traces in which there is no denaturation (Fig. S15).

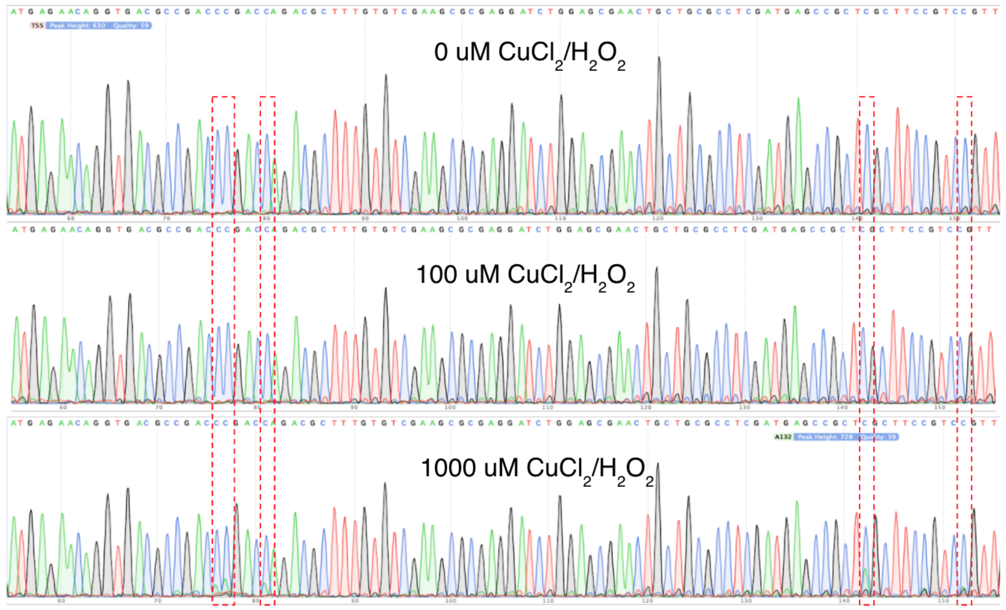

Figure S19: Characterization of the oxidation activity of $CuCl_2/H_2O_2$ by Sanger sequencing. The input was a 274 bp dsDNA oligo and was treated with different concentrations of $CuCl_2/H_2O_2$. The red boxes indicate where C->A mutations are detected when treated with 1000 μM $CuCl_2/H_2O_2$.

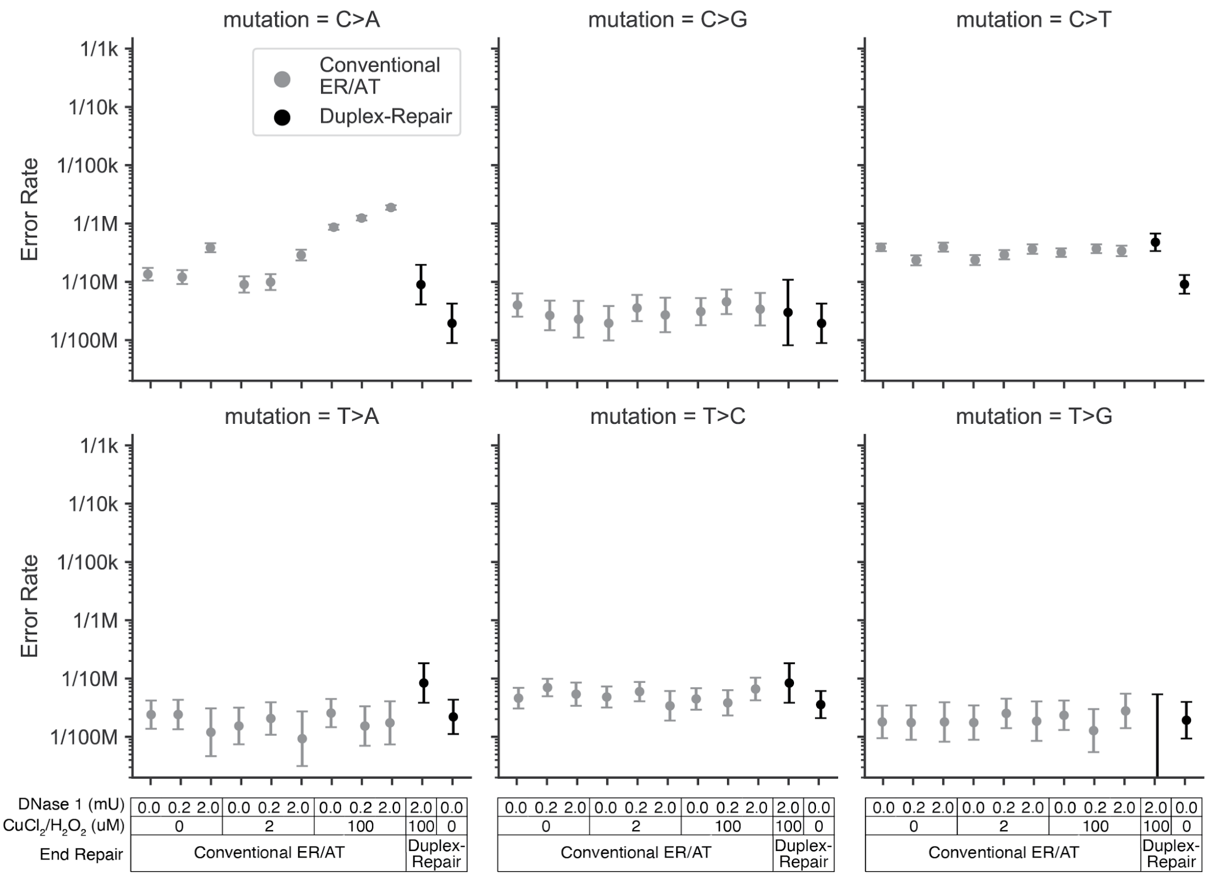

Figure S20: Error rates by mutation context observed in healthy donor cfDNA treated with varied concentrations of $CuCl_2/H_2O_2$ and DNase I.

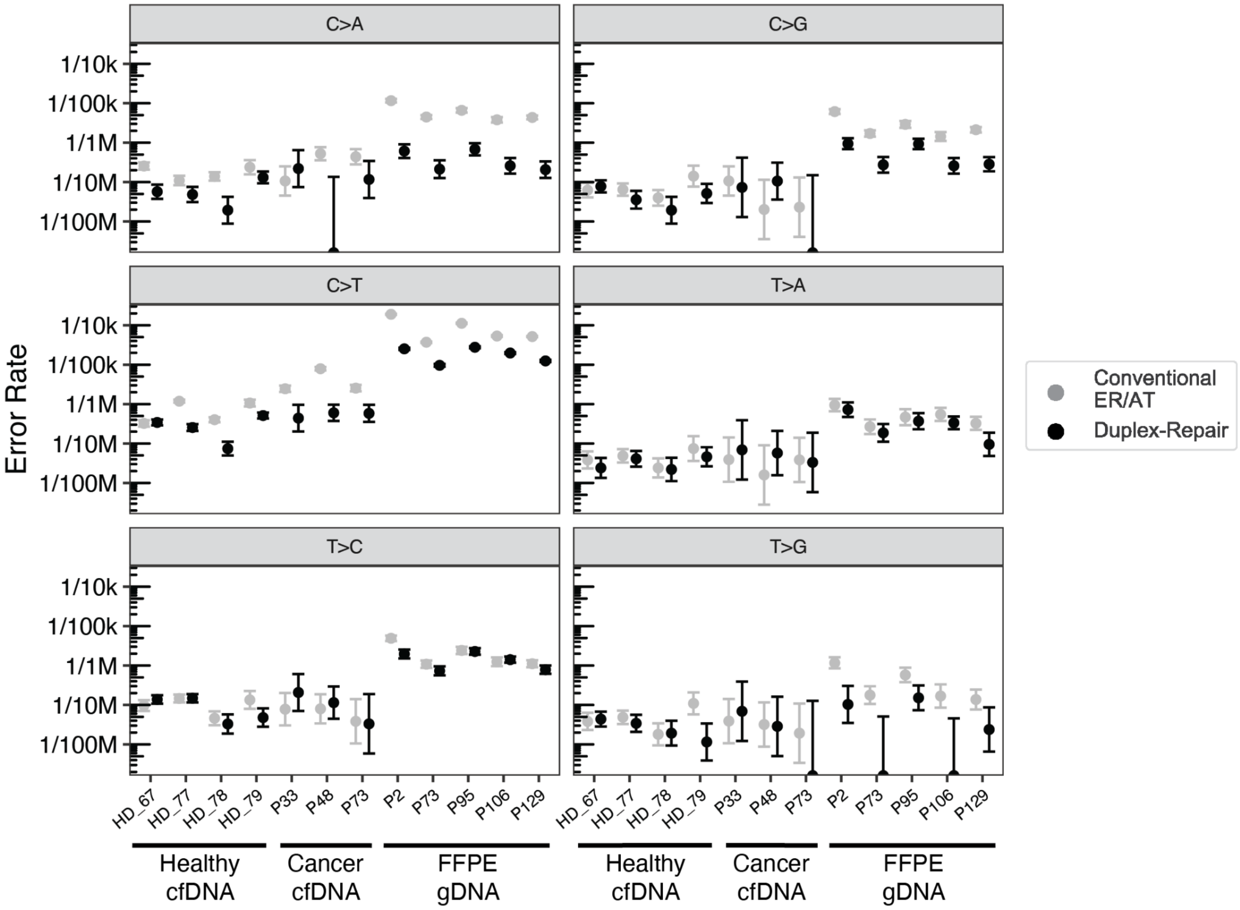

Figure S21: Error rates by mutation context observed in duplex sequencing of a pan-cancer panel for cfDNA samples and FFPE tumor biopsies treated with conventional ER/AT vs. Duplex-Repair.

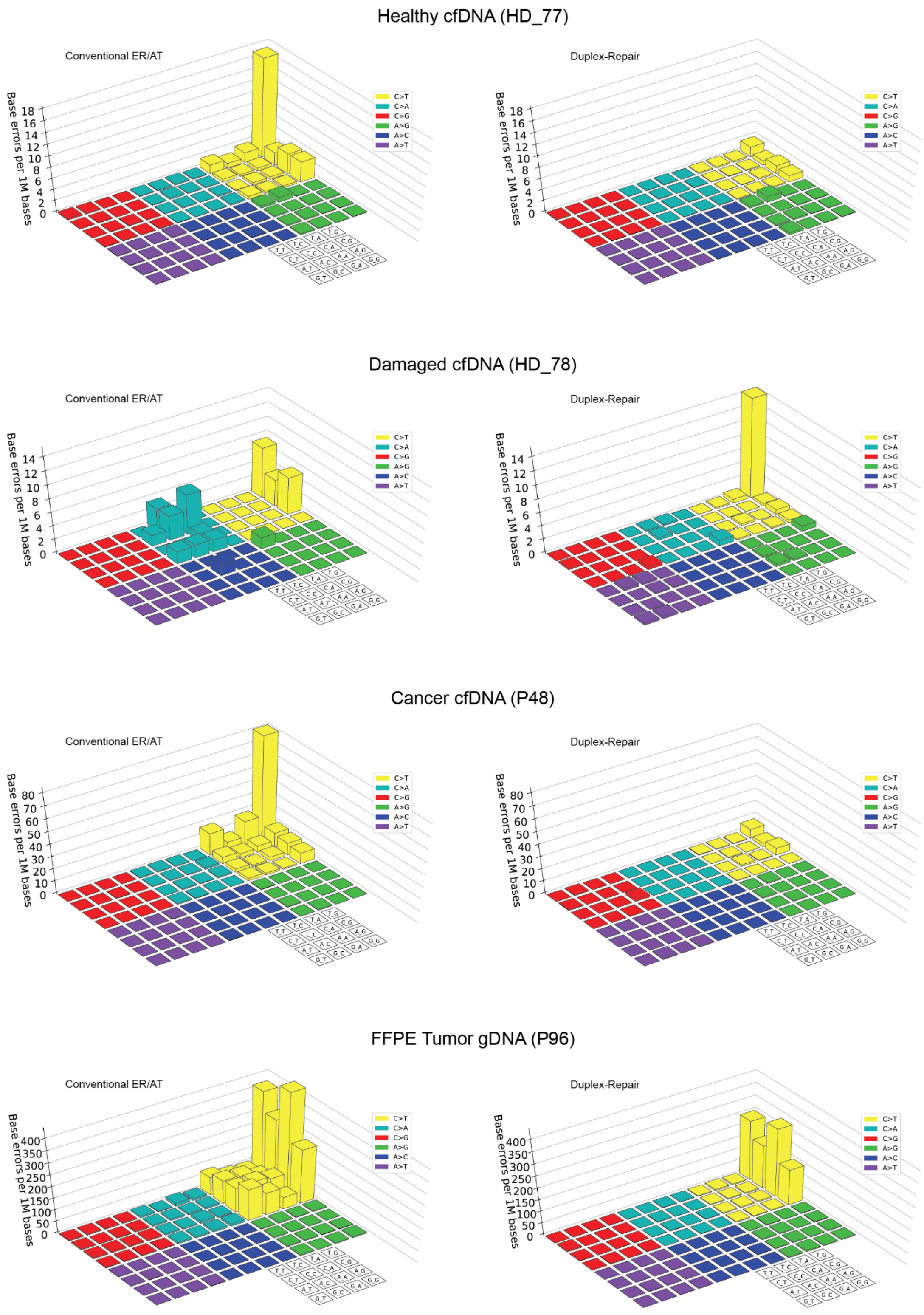

Figure S22: Base error rates per trinucleotide context. Tested for both conventional ER/AT and Duplex-Repair across healthy cfDNA, damaged cfDNA, cancer patient cfDNA, and FFPE tumor gDNA.

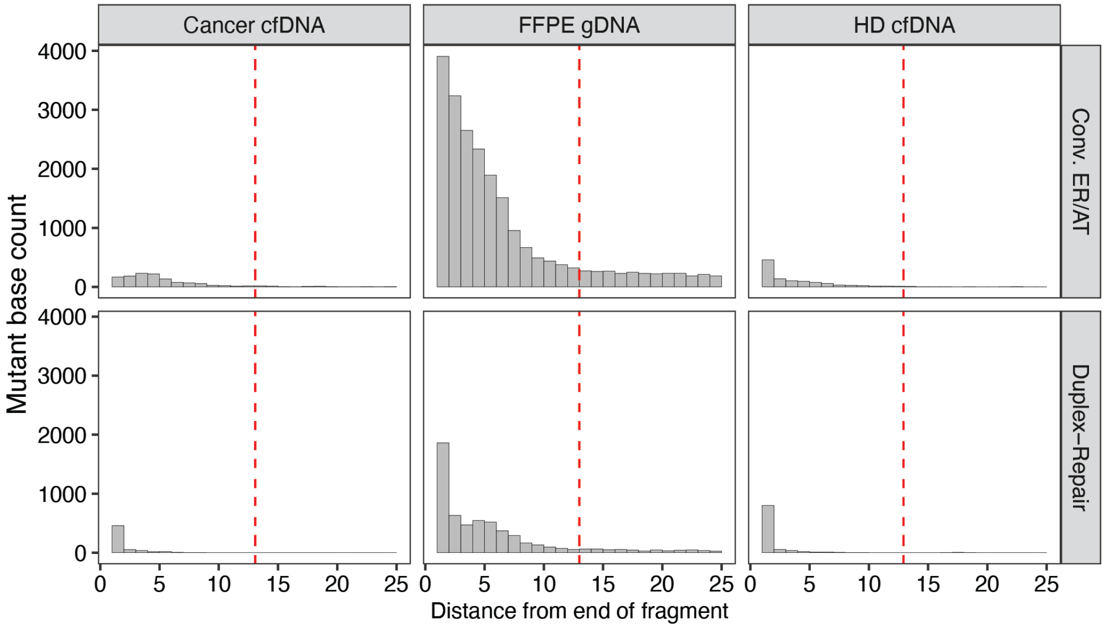

Figure S23: Distance of mutant duplex bases from closest DNA fragment end for cfDNA collected from healthy donors and cancer patients as well as gDNA from FFPE tumor biopsies. Samples underwent either conventional ER/AT or Duplex-Repair.

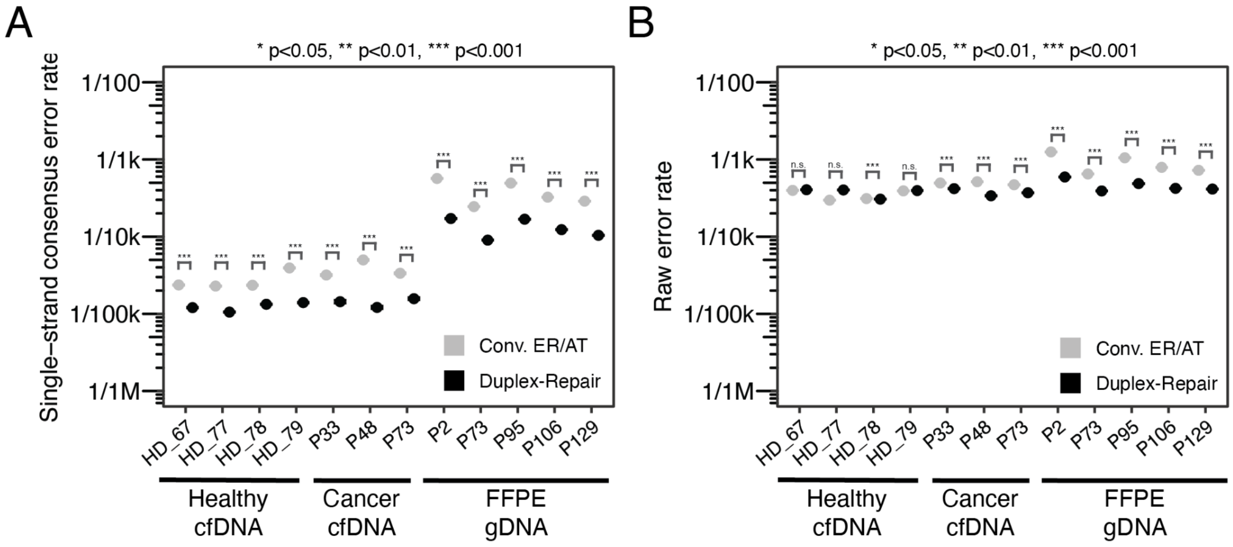

Figure S24: Single-strand consensus (A) and conventional raw NGS (B) error rates of four healthy cfDNA samples (three replicates per condition), three cancer patient cfDNA samples (one replicate per condition), and five cancer patient FFPE tumor biopsies (three replicates per condition) treated with conventional ER/AT or Duplex-Repair.

Table S1: DNA sequences of synthetic oligonucleotides used in this study. Asterisks (*) indicate the presence of a C3 spacer or phosphorothioate bonds that protect fluorophores from being cleaved by nucleases.

Oligo ID	Fluorophore end	Fluorophore	Sequence	Length (bp)
a	5'	6-FAM	6FAM//iSpC3/GCGTCACCAGCCACGCGAGCCGGATGAGGATCCGTGACGCGAAGT	48
b	5'	6-FAM	6FAM//iSpC3/GCGTCACCAGCCACGCGAGCCGGATGAGGATCCGTGACGCGAAGTCCTGGTACCGCCGCTCGCTTCCGAC	70
c	5'	6-FAM	6FAM//iSpC3/GCGTCACCAGCCACGCGAGCCGGATGAGGATCCGTGACGCGAAGTCCTGGTACCGCCGCTCGCTTCCGACCGGTTCTCCA	80
d	5'	6-FAM	6FAM//iSpC3/GCGTCACCAGCCACGCGAGCCGGATGAGGATCCGTGACGCGAAGTCCTGGTACCGCCGCTCGCTTCCGACCGGTTCTCCACCGAGCGACC	90
e	5'	6-FAM	6FAM//iSpC3/GCGTCACCAGCCACGCGAGCCGGATGAGGATCCGTGACGCGAAGTCCTGGTACCGCCGCTCGCTTCCGACCGGTTCTCCACCGAGCGACCTAATATTAAT	100
f	3'	ATTO 550	GTCGGAAGCGAGCGGCGGTACCAGGACTTCGCGTCACGGATCCTCATCCGGCTCGCGTGGCTGGTGACGC/iSpC3//3ATTO550	70
g	3'	ATTO 550	TGGAGAACCGGTCGGAAGCGAGCGGCGGTACCAGGACTTCGCGTCACGGATCCTCATCCGGCTCGCGTGGCTGGTGACGC/iSpC3//3ATTO550	80
h	3'	ATTO 550	GGTCGCTCGGTGGAGAACCGGTCGGAAGCGAGCGGCGGTACCAGGACTTCGCGTCACGGATCCTCATCCGGCTCGCGTGGCTGGTGACGC/iSpC3//3ATTO550	90
i	3'	ATTO 550	ATTAATATTAGGTCGCTCGGTGGAGAACCGGTCGGAAGCGAGCGGCGGTACCAGGACTTCGCGTCACGGATCCTCATCCGGCTCGCGTGGCTGGTG*A*C*G*C/3ATTO550 (*phosphorothioate	100

			bonds)	
j	3'	ATTO 550	ATTAATATTAGGTCGCTCGGTGGAGAACC/**<u>i8oxodG</u>**/GTCGGAAGCGAGCGGCGGTACCAGGACTTCGCGTCACGGATCCTCATCCGGCTCGCGTGGCTGGTGA*C*G*C*/3ATTO550N/ (*phosphorothioate bonds)	100
k	3'	ATTO 550	ATTAATATTAGGTCGCTCGGTGGAGAACC**<u>U</u>**GTCGGAAGCGAGCGGCGGTACCAGGACTTCGCGTCACGGATCCTCATCCGGCTCGCGTGGCTGGTGA*C*G*C*/3ATTO550N/ (*phosphorothioate bonds)	100
l	/	/	CGGTTCTCCACCGAGCGACCTAATATTAAT	30
m	/	/	GGTTCTCCACCGAGCGACCTAATATTAAT	29
n	/	/	CTCCACCGAGCGACCTAATATTAAT	25
o	/	/	GTCAAGGGTAATGGACAGTAGGTGTGGTGGAACATACTTCCAGCACTCAAGAAGCTGAAGCAGGCAGATCTCTGTCAGTTCATGACCACTGCTGTCTACATGGTGAGCTCCAAGCCAGCCAGGCAAGAAGTGACACTCAGGtCTCGCATTGCTcagACGgCaggcA	166
p	/	/	TgcctGcCGTctgAGCAATGCGAGaCCTGAGTGTCACTTCTTGCCTGGCTGGCTTGGAGCTCACCATGTAGACAGCAGTGGTCATGAACTGACAGAGATCTGCCTGCTTCAGCTTCTTGAGTGCTGGAAGTATGTTCCACCACACCTACTGTCCATTACCCTTGACA	166
q	/	/	GTCAAGGGTAATGGACAGTAGGTGTGGTGGAACATACTTCCAGCACTCAAGAAGCTGAAGCAGGCAGATCTCTGTCAGTTCATGACCACTGCTGTCTACATGGTGAGCTCCAAGCCAGCCAGGCAAGAAGTGACACTCAGGtCTCGCATTGCTcag	156
r	/	/	GTCAAGGGTAATGGACAGTAGGTGTGGTGGAACATACTTCCAGCACTCAAGAAGCTGAAGCAGGCAGATCTCTGTCAGTTCATGAC	86
s	/	/	CACTGCTGTCTACATGGTGAGCTCCAAGCCAGCCAGGCAAGAAGTGACACTCAGGtCTCGCATTGCTcagACGgCaggcA	80

t	/	/	ACTGCTGTCTACATGGTGAGCTCCAAGCCAG CCAGGCAAGAAGTGACACTCAGGtCTCGCATT GCTcagACGgCaggcA	79

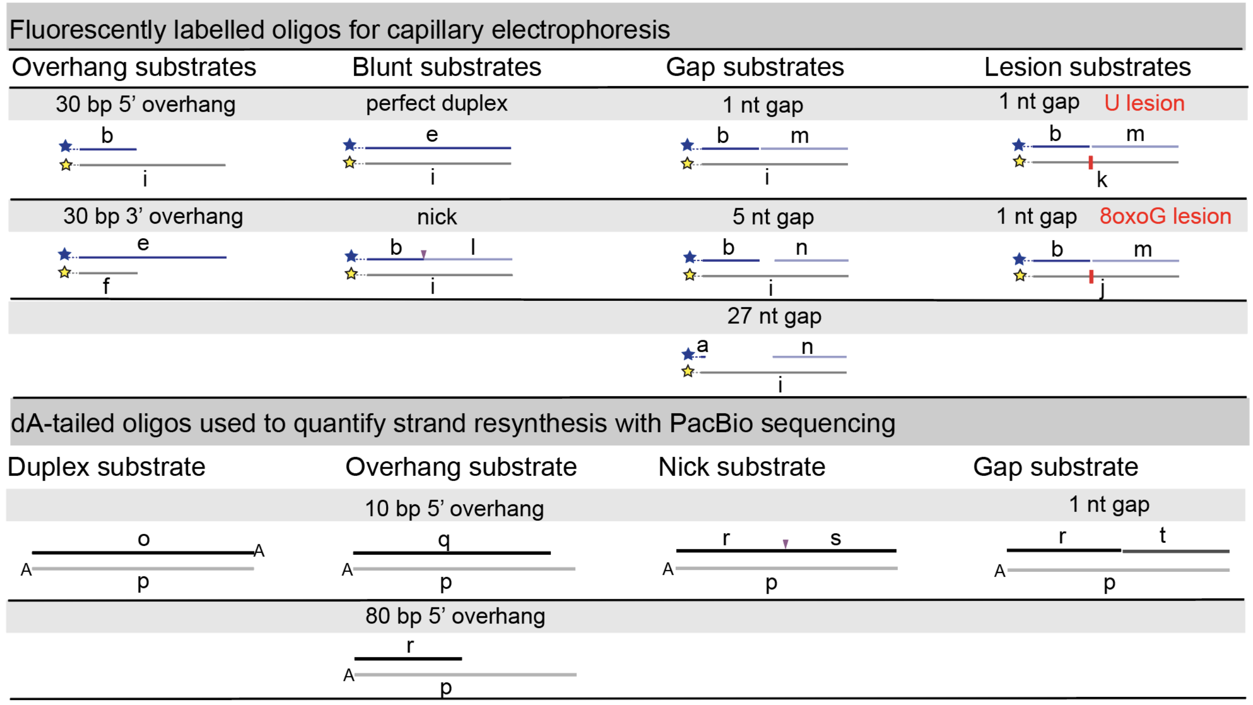

Table S2: Quantification of DNA loss after DNase 1 treatment. The input was 20 ng of a 100 bp dsDNA oligo. *the low yield indicates a significant loss during the Ampure bead cleanup step; ** the concentration of the 2nd biological replicate is below the detection limit of the Qubit assay.

Dnase I amount (mU)	Yields of DNA products after DNase 1 treatment, mean ± SD (with two biological replicates)
0	2.48 ± 0.32*
0.02	2.48 ± 0.04
0.2	2.18 ± 0.12
2	2.06 ± 0.04
20	1**

Table S3: Error rates and fold changes by mutation context for targeted panel sequencing. Duplex sequencing error rates broken down by mutation context for four healthy donor cfDNA samples, three cancer patient cfDNA samples, and five FFPE tumor biopsies. The samples were treated with Duplex-Repair and conventional ER/AT.

Specimen	Patient ID	Mutation Context	Error Rate Conventional ER/AT	Error Rate Duplex-Repair	Error Rate Fold Decrease	CI (95%)
cfDNA	HD_67	T>G	3.83E-08	4.38E-08	-1.14	[-2.06, -0.23]
cfDNA	HD_67	T>C	9.70E-08	1.38E-07	-1.42	[-2.29, -0.55]
cfDNA	HD_67	T>A	3.83E-08	2.41E-08	1.59	[0.89, 2.29]
cfDNA	HD_67	C>A	2.56E-07	5.71E-08	4.47	[3.98, 4.97]
cfDNA	HD_67	C>G	6.24E-08	7.79E-08	-1.25	[-2.13, -0.36]
cfDNA	HD_67	C>T	3.24E-07	3.43E-07	-1.06	[-2.04, -0.07]
cfDNA	HD_77	T>G	4.87E-08	3.42E-08	1.42	[0.63, 2.22]
cfDNA	HD_77	T>C	1.46E-07	1.48E-07	-1.01	[-2.01, -0.02]
cfDNA	HD_77	T>A	4.87E-08	4.11E-08	1.19	[0.28, 2.09]
cfDNA	HD_77	C>A	1.10E-07	4.85E-08	2.26	[1.61, 2.91]
cfDNA	HD_77	C>G	6.45E-08	3.57E-08	1.81	[1.12, 2.49]
cfDNA	HD_77	C>T	1.18E-06	2.55E-07	4.65	[3.93, 5.37]
cfDNA	HD_78	T>G	1.80E-08	1.93E-08	-1.07	[-2.01, -0.13]
cfDNA	HD_78	T>C	4.61E-08	3.30E-08	1.39	[0.60, 2.19]
cfDNA	HD_78	T>A	2.40E-08	2.20E-08	1.09	[0.17, 2.02]
cfDNA	HD_78	C>A	1.39E-07	1.94E-08	7.19	[7.00, 7.38]
cfDNA	HD_78	C>G	3.98E-08	1.94E-08	2.05	[1.54, 2.57]
cfDNA	HD_78	C>T	4.03E-07	7.43E-08	5.42	[4.94, 5.90]
cfDNA	HD_79	T>G	1.10E-07	1.15E-08	9.5	[9.44, 9.55]
cfDNA	HD_79	T>C	1.34E-07	4.79E-08	2.8	[2.34, 3.26]

cfDNA	HD_79	T>A	7.46E-08	4.61E-08	1.62	[0.98, 2.26]
cfDNA	HD_79	C>A	2.39E-07	1.31E-07	1.82	[1.09, 2.54]
cfDNA	HD_79	C>G	1.42E-07	5.15E-08	2.75	[2.32, 3.18]
cfDNA	HD_79	C>T	1.06E-06	5.13E-07	2.07	[1.25, 2.90]
cfDNA	P33	C>A	1.07E-07	2.20E-07	-2.06	[-2.42, -1.71]
cfDNA	P33	C>G	1.07E-07	7.34E-08	1.45	[1.00, 1.90]
cfDNA	P33	C>T	2.45E-06	4.40E-07	5.57	[5.32, 5.81]
cfDNA	P33	T>A	3.90E-08	6.92E-08	-1.77	[-2.03, -1.52]
cfDNA	P33	T>C	7.81E-08	2.08E-07	-2.66	[-2.89, -2.43]
cfDNA	P33	T>G	3.90E-08	6.92E-08	-1.77	[-2.03, -1.52]
cfDNA	P48	C>A	5.25E-07	0.00E+00	inf	[0.00, 0.00]
cfDNA	P48	C>G	2.02E-08	1.06E-07	-5.25	[-5.28, -5.23]
cfDNA	P48	C>T	7.89E-06	6.01E-07	13.13	[12.85, 13.42]
cfDNA	P48	T>A	1.60E-08	5.74E-08	-3.59	[-3.64, -3.54]
cfDNA	P48	T>C	7.99E-08	1.15E-07	-1.44	[-2.06, -0.82]
cfDNA	P48	T>G	3.20E-08	2.87E-08	1.11	[0.34, 1.89]
cfDNA	P73	C>A	4.39E-07	1.17E-07	3.77	[3.57, 3.96]
cfDNA	P73	C>G	2.31E-08	0.00E+00	inf	[0.00, 0.00]
cfDNA	P73	C>T	2.54E-06	5.83E-07	4.36	[3.91, 4.81]
cfDNA	P73	T>A	3.85E-08	3.32E-08	1.16	[0.46, 1.86]
cfDNA	P73	T>C	3.85E-08	3.32E-08	1.16	[0.46, 1.86]
cfDNA	P73	T>G	1.92E-08	0.00E+00	inf	[0.00, 0.00]
FFPE Biopsy	P106	C>A	3.80E-06	2.59E-07	14.68	[14.41, 14.95]
FFPE	P106	C>G	1.43E-06	2.59E-07	5.54	[5.14, 5.94]

Biopsy						
FFPE Biopsy	P106	C>T	5.33E-05	1.98E-05	2.7	[1.77, 3.63]
FFPE Biopsy	P106	T>A	5.48E-07	3.35E-07	1.64	[0.87, 2.40]
FFPE Biopsy	P106	T>C	1.24E-06	1.41E-06	-1.14	[-2.10, -0.17]
FFPE Biopsy	P106	T>G	1.69E-07	0.00E+00	inf	[0.00, 0.00]
FFPE Biopsy	P129	C>A	4.36E-06	2.07E-07	21.09	[20.87, 21.30]
FFPE Biopsy	P129	C>G	2.13E-06	2.84E-07	7.47	[7.07, 7.88]
FFPE Biopsy	P129	C>T	5.17E-05	1.25E-05	4.14	[3.24, 5.05]
FFPE Biopsy	P129	T>A	3.25E-07	9.53E-08	3.41	[3.03, 3.79]
FFPE Biopsy	P129	T>C	1.11E-06	7.87E-07	1.41	[0.52, 2.31]
FFPE Biopsy	P129	T>G	1.37E-07	2.38E-08	5.77	[5.70, 5.84]
FFPE Biopsy	P2	C>A	1.16E-05	6.10E-07	19.02	[18.72, 19.31]
FFPE Biopsy	P2	C>G	6.14E-06	9.41E-07	6.53	[6.01, 7.04]
FFPE Biopsy	P2	C>T	1.89E-04	2.53E-05	7.5	[6.63, 8.38]

FFPE Biopsy	P2	T>A	9.43E-07	7.24E-07	1.3	[0.44, 2.17]
FFPE Biopsy	P2	T>C	4.91E-06	1.96E-06	2.5	[1.74, 3.26]
FFPE Biopsy	P2	T>G	1.17E-06	1.03E-07	11.33	[11.27, 11.39]
FFPE Biopsy	P73	C>A	4.47E-06	2.13E-07	21.01	[20.81, 21.20]
FFPE Biopsy	P73	C>G	1.71E-06	2.74E-07	6.24	[5.84, 6.65]
FFPE Biopsy	P73	C>T	3.71E-05	9.63E-06	3.85	[2.96, 4.74]
FFPE Biopsy	P73	T>A	2.67E-07	1.86E-07	1.43	[0.65, 2.22]
FFPE Biopsy	P73	T>C	1.08E-06	7.32E-07	1.47	[0.60, 2.35]
FFPE Biopsy	P73	T>G	1.78E-07	0.00E+00	inf	[0.00, 0.00]
FFPE Biopsy	P95	C>A	6.60E-06	6.79E-07	9.73	[9.31, 10.15]
FFPE Biopsy	P95	C>G	2.91E-06	9.28E-07	3.14	[2.48, 3.80]
FFPE Biopsy	P95	C>T	1.12E-04	2.77E-05	4.05	[3.14, 4.97]
FFPE Biopsy	P95	T>A	4.67E-07	3.69E-07	1.27	[0.41, 2.12]
FFPE	P95	T>C	2.42E-06	2.28E-06	1.06	[0.08, 2.05]

Biopsy						
FFPE Biopsy	P95	T>G	5.77E-07	1.52E-07	3.8	[3.48, 4.12]

Table S4: Sequencing metrics for all samples profiled by targeted panel sequencing.

Specimen	Patient ID	DNA damage inducers	ER/AT method	Number of raw reads	On target rates	Number of duplex bases evaluated	Number of base errors	Error Rate	CI (95%)
cfDNA	HD_78	0μM_CuCl2/ H2O2+0mU_ DNase1	Conv. ER/AT	1.29E+08	0.98172	12216528	1	8.19E-08	[1.44e-08, 4.64e-07]
cfDNA	HD_78	0μM_CuCl2/ H2O2+0.2mU _DNase1	Conv. ER/AT	1.54E+08	0.982728	10417986	3	2.88E-07	[9.79e-08, 8.47e-07]
cfDNA	HD_78	0μM_CuCl2/ H2O2+2mU_ DNase1	Conv. ER/AT	1.39E+08	0.983843	9406423	5	5.32E-07	[2.27e-07, 1.24e-06]
cfDNA	HD_78	2μM_CuCl2/ H2O2+0mU_ DNase1	Conv. ER/AT	1.58E+08	0.982447	10542247	4	3.79E-07	[1.48e-07, 9.76e-07]
cfDNA	HD_78	2μM_CuCl2/ H2O2+0.2mU _DNase1	Conv. ER/AT	1.36E+08	0.982074	11439892	5	4.37E-07	[1.87e-07, 1.02e-06]

cfDNA	HD_78	2μM_CuCl2/H2O2+2mU_DNase1	Conv. ER/AT	1.85E+08	0.983726	7559620	5	6.61E-07	[2.83e-07, 1.55e-06]
cfDNA	HD_78	100μM_CuCl2/H2O2+0mU_DNase1	Conv. ER/AT	1.33E+08	0.982054	11668760	5	4.28E-07	[1.83e-07, 1.00e-06]
cfDNA	HD_78	100μM_CuCl2/H2O2+0.2mU_DNase1	Conv. ER/AT	1.47E+08	0.981823	10624035	7	6.59E-07	[3.19e-07, 1.36e-06]
cfDNA	HD_78	100μM_CuCl2/H2O2+2mU_DNase1	Conv. ER/AT	1.66E+08	0.983928	8380284	7	8.35E-07	[4.05e-07, 1.72e-06]
cfDNA	HD_78	0μM_CuCl2/H2O2+0mU_DNase1	Conv. ER/AT	1.51E+08	0.981821	13171944	3	2.28E-07	[7.75e-08, 6.70e-07]
cfDNA	HD_78	0μM_CuCl2/H2O2+0.2mU_DNase1	Conv. ER/AT	1.30E+08	0.982407	11141949	3	2.69E-07	[9.16e-08, 7.92e-07]
cfDNA	HD_78	0μM_CuCl2/H2O2+2mU_DNase1	Conv. ER/AT	1.66E+08	0.983527	10042644	6	5.97E-07	[2.74e-07, 1.30e-06]
cfDNA	HD_78	2μM_CuCl2/H2O2+0mU_DNase1	Conv. ER/AT	1.20E+08	0.982558	10956968	1	9.13E-08	[1.61e-08, 5.17e-07]
cfDNA	HD_78	2μM_CuCl2/H2O2+0.2mU_DNase1	Conv. ER/AT	1.67E+08	0.982041	10056176	2	1.99E-07	[5.45e-08, 7.25e-07]
cfDNA	HD_78	2μM_CuCl2/	Conv.	1.95E	0.983	89659	3	3.35E	[1.14e

		H2O2+2mU_DNase1	ER/AT	+08	867	81		-07	-07, 9.84e-07]
cfDNA	HD_78	100μM_CuCl2/H2O2+0mU_DNase1	Conv. ER/AT	1.30E+08	0.982483	12904331	3	2.32E-07	[7.91e-08, 6.84e-07]
cfDNA	HD_78	100μM_CuCl2/H2O2+0.2mU_DNase1	Conv. ER/AT	1.30E+08	0.981903	10159501	6	5.91E-07	[2.71e-07, 1.29e-06]
cfDNA	HD_78	100μM_CuCl2/H2O2+2mU_DNase1	Conv. ER/AT	1.52E+08	0.984147	6953914	7	1.01E-06	[4.88e-07, 2.08e-06]
cfDNA	HD_78	0μM_CuCl2/H2O2+0mU_DNase1	Conv. ER/AT	1.49E+08	0.980883	12742674	4	3.14E-07	[1.22e-07, 8.07e-07]
cfDNA	HD_78	0μM_CuCl2/H2O2+0.2mU_DNase1	Conv. ER/AT	1.39E+08	0.981531	12832585	3	2.34E-07	[7.95e-08, 6.87e-07]
cfDNA	HD_78	0μM_CuCl2/H2O2+2mU_DNase1	Conv. ER/AT	1.48E+08	0.983953	6998068	2	2.86E-07	[7.84e-08, 1.04e-06]
cfDNA	HD_78	2μM_CuCl2/H2O2+0mU_DNase1	Conv. ER/AT	1.53E+08	0.981475	13027274	0	0	[0.00e+00, 2.95e-07]
cfDNA	HD_78	2μM_CuCl2/H2O2+0.2mU_DNase1	Conv. ER/AT	1.54E+08	0.982044	11278606	1	8.87E-08	[1.57e-08, 5.02e-07]
cfDNA	HD_78	2μM_CuCl2/H2O2+2mU_	Conv. ER/AT	1.82E+08	0.982618	8682012	5	5.76E-07	[2.46e-07,

		DNase1							1.35e-06]
cfDNA	HD_78	100µM_CuCl2/H2O2+0mU_DNase1	Conv. ER/AT	1.37E+08	0.981088	11267015	5	4.44E-07	[1.90e-07, 1.04e-06]
cfDNA	HD_78	100µM_CuCl2/H2O2+0.2mU_DNase1	Conv. ER/AT	1.33E+08	0.980935	8874034	3	3.38E-07	[1.15e-07, 9.94e-07]
cfDNA	HD_78	100µM_CuCl2/H2O2+2mU_DNase1	Conv. ER/AT	1.27E+08	0.983378	7319479	5	6.83E-07	[2.92e-07, 1.60e-06]
cfDNA	damaged_HD_78_cfDNA	100µM_CuCl2/H2O2_+_2mU_DNase1	Duplex-Repair	5.34E+07	0.988432	44538494	13	2.92E-07	[1.71e-07, 4.99e-07]
cfDNA	damaged_HD_78_cfDNA	100µM_CuCl2/H2O2_+_2mU_DNase2	Duplex-Repair	6.42E+07	0.988659	43911733	21	4.78E-07	[3.13e-07, 7.31e-07]
cfDNA	damaged_HD_78_cfDNA	100µM_CuCl2/H2O2_+_2mU_DNase3	Duplex-Repair	6.92E+07	0.988647	50237066	18	3.58E-07	[2.27e-07, 5.66e-07]
cfDNA	HD_78	NA	Duplex-Repair	1.12E+08	0.983891	220967435	20	9.05E-08	[5.86e-08, 1.40e-07]
cfDNA	HD_78	NA	Duplex-Repair	1.22E+08	0.982716	205270556	24	1.17E-07	[7.86e-08, 1.74e-07]
cfDNA	HD_78	NA	Duplex-Repair	1.15E+08	0.983438	246649702	24	9.73E-08	[6.54e-08, 1.45e

									-07]
cfDNA	P33	NA	Duple x- Repair	1.48E +07	0.965 585	28090 417	15	5.34E -07	[3.24e -07, 8.81e -07]
cfDNA	P33	NA	Conv. ER/AT	3.96E +07	0.970 814	98148 695	133	1.36E -06	[1.14e -06, 1.61e -06]
cfDNA	P48	NA	Duple x- Repair	3.91E +07	0.986 819	63145 388	27	4.28E -07	[2.94e -07, 6.22e -07]
cfDNA	P48	NA	Conv. ER/AT	6.12E +07	0.980 437	11212 2125	426	3.80E -06	[3.46e -06, 4.18e -06]
cfDNA	P73	NA	Duple x- Repair	3.39E +07	0.983 176	55844 344	20	3.58E -07	[2.32e -07, 5.53e -07]
cfDNA	P73	NA	Conv. ER/AT	3.46E +07	0.981 357	95264 860	135	1.42E -06	[1.20e -06, 1.68e -06]
FFPE Tumor Biopsy	P106	NA	Conv. ER/AT	5.36E +07	0.988 84	20006 860	521	2.60E -05	[2.39e -05, 2.84e -05]
FFPE Tumor Biopsy	P106	NA	Conv. ER/AT	6.61E +07	0.988 419	23707 349	665	2.81E -05	[2.60e -05, 3.03e -05]
FFPE Tumor Biopsy	P106	NA	Conv. ER/AT	9.51E +07	0.988 755	43482 673	1235	2.84E -05	[2.69e -05, 3.00e -05]

FFPE Tumor Biopsy	P106	NA	Duplex-Repair	6.73E+07	0.991287	49319461	467	9.47E-06	[8.65e-06, 1.04e-05]
FFPE Tumor Biopsy	P106	NA	Duplex-Repair	8.13E+07	0.991346	53118736	538	1.01E-05	[9.31e-06, 1.10e-05]
FFPE Tumor Biopsy	P106	NA	Duplex-Repair	6.85E+07	0.991322	50713014	551	1.09E-05	[9.99e-06, 1.18e-05]
FFPE Tumor Biopsy	P95	NA	Conv. ER/AT	8.51E+07	0.990015	17146216	879	5.13E-05	[4.80e-05, 5.48e-05]
FFPE Tumor Biopsy	P95	NA	Conv. ER/AT	7.61E+07	0.990807	24648513	1564	6.35E-05	[6.04e-05, 6.67e-05]
FFPE Tumor Biopsy	P95	NA	Conv. ER/AT	8.33E+07	0.990089	27917450	1734	6.21E-05	[5.93e-05, 6.51e-05]
FFPE Tumor Biopsy	P95	NA	Duplex-Repair	8.51E+07	0.991906	32413963	530	1.64E-05	[1.50e-05, 1.78e-05]
FFPE Tumor Biopsy	P95	NA	Duplex-Repair	7.40E+07	0.991789	28687673	471	1.64E-05	[1.50e-05, 1.80e-05]
FFPE Tumor Biopsy	P95	NA	Duplex-Repair	7.44E+07	0.991882	29219082	421	1.44E-05	[1.31e-05, 1.59e-05]
FFPE	P2	NA	Conv.	6.47E	0.984	13700	1078	7.87E	[7.41e

Tumor Biopsy			ER/AT	+07	017	238		-05	-05, 8.35e -05]
FFPE Tumor Biopsy	P2	NA	Conv. ER/AT	5.20E +07	0.985 261	23873 009	2729	0.000 11431	[1.10e -04, 1.19e -04]
FFPE Tumor Biopsy	P2	NA	Conv. ER/AT	7.87E +07	0.986 823	24256 287	2853	0.000 11762	[1.13e -04, 1.22e -04]
FFPE Tumor Biopsy	P2	NA	Duple x- Repair	1.00E +08	0.992 637	26565 686	469	1.77E -05	[1.61e -05, 1.93e -05]
FFPE Tumor Biopsy	P2	NA	Duple x- Repair	1.03E +08	0.992 481	27540 728	457	1.66E -05	[1.51e -05, 1.82e -05]
FFPE Tumor Biopsy	P2	NA	Duple x- Repair	9.27E +07	0.992 096	14224 743	209	1.47E -05	[1.28e -05, 1.68e -05]
FFPE Tumor Biopsy	P73	NA	Conv. ER/AT	9.77E +07	0.988 559	49482 848	988	2.00E -05	[1.88e -05, 2.13e -05]
FFPE Tumor Biopsy	P73	NA	Conv. ER/AT	8.96E +07	0.988 492	45128 942	860	1.91E -05	[1.78e -05, 2.04e -05]
FFPE Tumor Biopsy	P73	NA	Conv. ER/AT	8.27E +07	0.991 062	51361 631	1184	2.31E -05	[2.18e -05, 2.44e -05]
FFPE Tumor	P73	NA	Duple x-	9.48E +07	0.991 359	46828 201	251	5.36E -06	[4.74e -06,

Biopsy			Repair						6.07e-06]
FFPE Tumor Biopsy	P73	NA	Duplex-Repair	9.98E+07	0.992118	43268486	197	4.55E-06	[3.96e-06, 5.23e-06]
FFPE Tumor Biopsy	P73	NA	Duplex-Repair	1.03E+08	0.991284	50748145	286	5.64E-06	[5.02e-06, 6.33e-06]
FFPE Tumor Biopsy	P129	NA	Conv. ER/AT	6.19E+07	0.990651	38198285	790	2.07E-05	[1.93e-05, 2.22e-05]
FFPE Tumor Biopsy	P129	NA	Conv. ER/AT	5.70E+07	0.988617	46259759	1411	3.05E-05	[2.90e-05, 3.21e-05]
FFPE Tumor Biopsy	P129	NA	Conv. ER/AT	8.37E+07	0.98878	67552550	2111	3.12E-05	[2.99e-05, 3.26e-05]
FFPE Tumor Biopsy	P129	NA	Duplex-Repair	6.32E+07	0.990994	46410054	300	6.46E-06	[5.77e-06, 7.24e-06]
FFPE Tumor Biopsy	P129	NA	Duplex-Repair	6.47E+07	0.990846	47452054	315	6.64E-06	[5.94e-06, 7.41e-06]
FFPE Tumor Biopsy	P129	NA	Duplex-Repair	6.50E+07	0.991667	67397721	463	6.87E-06	[6.27e-06, 7.52e-06]
cfDNA	HD_67	NA	Conv. ER/AT	1.15E+08	0.987253	287241318	94	3.27E-07	[2.67e-07, 4.00e

									-07]
cfDNA	HD_67	NA	Conv. ER/AT	1.21E+08	0.987747	228373435	100	4.38E-07	[3.60e-07, 5.33e-07]
cfDNA	HD_67	NA	Conv. ER/AT	1.07E+08	0.987641	212510412	90	4.24E-07	[3.45e-07, 5.21e-07]
cfDNA	HD_67	NA	Duplex-Repair	155620562	0.980312	326796016	95	2.91E-07	[2.38e-07, 3.55e-07]
cfDNA	HD_67	NA	Duplex-Repair	113886380	0.980136	242483444	102	4.21E-07	[3.47e-07, 5.11e-07]
cfDNA	HD_67	NA	Duplex-Repair	127505618	0.980876	272308087	81	2.97E-07	[2.39e-07, 3.70e-07]
cfDNA	HD_77	NA	Conv. ER/AT	159711982	0.987585	459509299	318	6.92E-07	[6.20e-07, 7.72e-07]
cfDNA	HD_77	NA	Conv. ER/AT	134944370	0.988378	247081170	201	8.13E-07	[7.09e-07, 9.34e-07]
cfDNA	HD_77	NA	Conv. ER/AT	126086546	0.987581	271681550	238	8.76E-07	[7.72e-07, 9.95e-07]
cfDNA	HD_77	NA	Duplex-Repair	168105150	0.976197	320539624	96	2.99E-07	[2.45e-07, 3.66e-07]

cfDNA	HD_77	NA	Duplex-Repair	147197612	0.97676	287680401	74	2.57E-07	[2.05e-07, 3.23e-07]
cfDNA	HD_77	NA	Duplex-Repair	113851702	0.973208	222307450	61	2.74E-07	[2.14e-07, 3.52e-07]
cfDNA	HD_79	NA	Conv. ER/AT	83749956	0.978007	20839707	26	1.25E-06	[8.51e-07, 1.83e-06]
cfDNA	HD_79	NA	Conv. ER/AT	89172830	0.977934	22898460	25	1.09E-06	[7.40e-07, 1.61e-06]
cfDNA	HD_79	NA	Conv. ER/AT	146134132	0.983414	152767879	109	7.14E-07	[5.92e-07, 8.61e-07]
cfDNA	HD_79	NA	Duplex-Repair	178781140	0.977662	251559928	88	3.50E-07	[2.84e-07, 4.31e-07]
cfDNA	HD_79	NA	Duplex-Repair	90429248	0.974458	22015921	35	1.59E-06	[1.14e-06, 2.21e-06]
cfDNA	HD_79	NA	Duplex-Repair	160665758	0.977917	241459115	74	3.06E-07	[2.44e-07, 3.85e-07]

Table S5: Clinical samples tested with calculated p-values.

patient_id	dna_type	data_type	total_base_errors_er-at	total_bases_eval_er-at	total_base_errors_d-r	total_bases_eval_d-r	binom_p_value	stars
P106	gDNA	dsc	2421	87196882	1.56E+03	1.53E+08	1.26E-228	***
P129	gDNA	dsc	4312	152010594	1.08E+03	1.61E+08	0	***
P2	gDNA	dsc	6660	61829534	1.14E+03	6.83E+07	0	***
P33	cfDNA	dsc	133	98148695	1.50E+01	2.81E+07	0.0001971	***
P48	cfDNA	dsc	426	112122125	27	63145388	7.73E-41	***
P73	cfDNA	dsc	135	95264860	20	55844344	2.75E-10	***
P73	gDNA	dsc	3032	145973421	734	140844832	1.09E-289	***
P95	gDNA	dsc	4177	69712179	1422	90320718	0	***
HD_67	cfDNA	dsc	284	728125165	278	841587547	0.02431536	*
HD_77	cfDNA	dsc	757	978272019	231	830527475	3.73E-46	***
HD_78	cfDNA	dsc	307	951472269	62	672887687	3.92E-22	***
HD_79	cfDNA	dsc	160	196506046	197	515034964	1.81E-13	***
HD_67	cfDNA	ssc	64515	2726357147	44325	3682679397	0	***
HD_77	cfDNA	ssc	77658	3393231581	33436	3175674821	0	***
HD_78	cfDNA	ssc	62391	2659902	40531	3047703	0	***

				925		136		
HD_79	cfDNA	ssc	38706	983475008	27059	1933927680	0	***
P106	gDNA	ssc	253906	781258501	115514	937318233	0	***
P129	gDNA	ssc	269950	935738709	96514	926947295	0	***
P2	gDNA	ssc	246281	433978486	70662	411190318	0	***
P33	cfDNA	ssc	8907	279873419	1989	138474936	3.81E-238	***
P48	cfDNA	ssc	17187	344001755	2633	217476334	0	***
P73	cfDNA	ssc	10818	322502770	3476	221179942	0	***
P73	gDNA	ssc	254718	1038992250	89271	989954527	0	***
P95	gDNA	ssc	259101	524568382	83345	495358454	0	***
HD_67	cfDNA	raw	1078421	2710271751	1629802	4018288526	1	-
HD_77	cfDNA	raw	928213	3127576014	1283583	3184355471	1	-
HD_78	cfDNA	raw	1136603	3648579542	957481	3126690908	2.39E-35	***
HD_79	cfDNA	raw	407005	1037374537	741377	1875556018	0.99993612	-
P106	gDNA	raw	864788	1093812190	482096	1140806327	0	***
P129	gDNA	raw	824944	1139213367	449896	1086832587	0	***
P2	gDNA	raw	747902	595871040	295360	498127821	0	***

P33	cfDNA	raw	131725	265718883	76541	182637255	4.60E-300	***
P48	cfDNA	raw	178553	346978490	76638	226252117	0	***
P73	cfDNA	raw	155983	331482592	88822	239767778	0	***
P73	gDNA	raw	831924	1281655767	462957	1183109503	0	***
P95	gDNA	raw	727896	692246025	284807	584619745	0	***
0uM_CuCl2/H2O2+0mU_DNase1	cfDNA	dsc	307	951472269	68	672887693	2.68E-20	***
100uM_CuCl2/H2O2+2mU_DNase1	cfDNA	dsc	648	553553932	5.20E+01	138687293	3.95E-17	***

Table S6: Error rates for SSC and conventional raw NGS data.

Specimen	Patient ID	Data Type	ER/AT method	Number of bases evaluated	Number of base errors	Error Rate	CI (95%)
cfDNA	HD_67	raw	Conv. ER/AT	2710271751	1.08E+06	3.98E-04	[3.97e-04, 3.99e-04]
cfDNA	HD_67	raw	Duplex-Repair	4018288526	1.63E+06	4.06E-04	[4.05e-04, 4.06e-04]
cfDNA	HD_77	raw	Conv. ER/AT	3127576014	9.28E+05	2.97E-04	[2.96e-04, 2.97e-04]
cfDNA	HD_77	raw	Duplex-Repair	3184355471	1.28E+06	4.03E-04	[4.02e-04, 4.04e-04]
cfDNA	HD_78	raw	Conv. ER/AT	3648579542	1136603	3.12E-04	[3.11e-04, 3.12e-04]

cfDNA	HD_78	raw	Duplex-Repair	3126690908	957481	3.06E-04	[3.06e-04, 3.07e-04]
cfDNA	HD_79	raw	Conv. ER/AT	1037374537	407005	3.92E-04	[3.91e-04, 3.94e-04]
cfDNA	HD_79	raw	Duplex-Repair	1875556018	741377	3.95E-04	[3.94e-04, 3.96e-04]
gDNA	P106	raw	Conv. ER/AT	1093812190	864788	7.91E-04	[7.89e-04, 7.92e-04]
gDNA	P106	raw	Duplex-Repair	1140806327	482096	4.23E-04	[4.21e-04, 4.24e-04]
gDNA	P129	raw	Conv. ER/AT	1139213367	824944	7.24E-04	[7.23e-04, 7.26e-04]
gDNA	P129	raw	Duplex-Repair	1086832587	449896	4.14E-04	[4.13e-04, 4.15e-04]
gDNA	P2	raw	Conv. ER/AT	595871040	747902	1.26E-03	[1.25e-03, 1.26e-03]
gDNA	P2	raw	Duplex-Repair	498127821	295360	5.93E-04	[5.91e-04, 5.95e-04]
cfDNA	P33	raw	Conv. ER/AT	265718883	131725	4.96E-04	[4.93e-04, 4.98e-04]
cfDNA	P33	raw	Duplex-Repair	182637255	76541	4.19E-04	[4.16e-04, 4.22e-04]
cfDNA	P48	raw	Conv. ER/AT	346978490	178553	5.15E-04	[5.12e-04, 5.17e-04]
cfDNA	P48	raw	Duplex-Repair	226252117	76638	3.39E-04	[3.36e-04, 3.41e-04]
cfDNA	P73	raw	Conv. ER/AT	331482592	155983	4.71E-04	[4.68e-04, 4.73e-04]
cfDNA	P73	raw	Duplex-Repair	239767778	88822	3.70E-04	[3.68e-04, 3.73e-04]
gDNA	P73	raw	Conv. ER/AT	1281655767	831924	6.49E-04	[6.48e-04, 6.50e-04]

gDNA	P73	raw	Duplex-Repair	1183109503	462957	3.91E-04	[3.90e-04, 3.92e-04]
gDNA	P95	raw	Conv. ER/AT	692246025	727896	1.05E-03	[1.05e-03, 1.05e-03]
gDNA	P95	raw	Duplex-Repair	584619745	284807	4.87E-04	[4.85e-04, 4.89e-04]
cfDNA	HD_67	ssc	Conv. ER/AT	2726357147	64515	2.37E-05	[2.35e-05, 2.38e-05]
cfDNA	HD_67	ssc	Duplex-Repair	3682679397	44325	1.20E-05	[1.19e-05, 1.21e-05]
cfDNA	HD_77	ssc	Conv. ER/AT	3393231581	77658	2.29E-05	[2.27e-05, 2.30e-05]
cfDNA	HD_77	ssc	Duplex-Repair	3175674821	33436	1.05E-05	[1.04e-05, 1.06e-05]
cfDNA	HD_78	ssc	Conv. ER/AT	2659902925	62391	2.35E-05	[2.33e-05, 2.36e-05]
cfDNA	HD_78	ssc	Duplex-Repair	3047703136	40531	1.33E-05	[1.32e-05, 1.34e-05]
cfDNA	HD_79	ssc	Conv. ER/AT	983475008	38706	3.94E-05	[3.90e-05, 3.98e-05]
cfDNA	HD_79	ssc	Duplex-Repair	1933927680	27059	1.40E-05	[1.38e-05, 1.42e-05]
gDNA	P106	ssc	Conv. ER/AT	781258501	253906	3.25E-04	[3.24e-04, 3.26e-04]
gDNA	P106	ssc	Duplex-Repair	937318233	115514	1.23E-04	[1.23e-04, 1.24e-04]
gDNA	P129	ssc	Conv. ER/AT	935738709	269950	2.88E-04	[2.87e-04, 2.90e-04]
gDNA	P129	ssc	Duplex-Repair	926947295	96514	1.04E-04	[1.03e-04, 1.05e-04]
gDNA	P2	ssc	Conv. ER/AT	433978486	246281	5.67E-04	[5.65e-04, 5.70e-04]

gDNA	P2	ssc	Duplex-Repair	411190318	70662	1.72E-04	[1.71e-04, 1.73e-04]
cfDNA	P33	ssc	Conv. ER/AT	279873419	8.91E+03	3.18E-05	[3.12e-05, 3.25e-05]
cfDNA	P33	ssc	Duplex-Repair	138474936	1.99E+03	1.44E-05	[1.37e-05, 1.50e-05]
cfDNA	P48	ssc	Conv. ER/AT	344001755	17187	5.00E-05	[4.92e-05, 5.07e-05]
cfDNA	P48	ssc	Duplex-Repair	217476334	2633	1.21E-05	[1.17e-05, 1.26e-05]
cfDNA	P73	ssc	Conv. ER/AT	322502770	10818	3.35E-05	[3.29e-05, 3.42e-05]
cfDNA	P73	ssc	Duplex-Repair	221179942	3476	1.57E-05	[1.52e-05, 1.62e-05]
gDNA	P73	ssc	Conv. ER/AT	1038992250	254718	2.45E-04	[2.44e-04, 2.46e-04]
gDNA	P73	ssc	Duplex-Repair	989954527	89271	9.02E-05	[8.96e-05, 9.08e-05]
gDNA	P95	ssc	Conv. ER/AT	524568382	259101	4.94E-04	[4.92e-04, 4.96e-04]
gDNA	P95	ssc	Duplex-Repair	495358454	83345	1.68E-04	[1.67e-04, 1.69e-04]
